# The prognostic effect of tumor volume, reduction ratio, and cumulative doses on external beam radiotherapy with central-shielding method and image-guided adaptive brachytherapy for cervical cancer

**DOI:** 10.3389/fonc.2024.1366777

**Published:** 2024-05-07

**Authors:** Takeru Ohtaka, Ken Ando, Takahiro Oike, Shin-ei Noda, Takuya Kaminuma, Kazutoshi Murata, Tatsuya Ohno

**Affiliations:** ^1^ Department of Radiation Oncology, Gunma University Graduate School of Medicine, Maebashi, Japan; ^2^ Gunma University Heavy Ion Medical Center, Maebashi, Japan; ^3^ Department of Radiation Oncology, Saitama Medical University International Medical Center, Hidaka, Japan; ^4^ Department of Radiation Therapy, NHO Shibukawa Medical Center, Shibukawa, Japan; ^5^ QST Hospital, National Institutes for Quantum and Radiological Science and Technology, Chiba, Japan

**Keywords:** cervical cancer, radiotherapy, brachytherapy, prognosis, tumor volume, reduction ratio, cumulative doses

## Abstract

**Objective:**

To evaluate the prognostic effect of tumor volume at diagnosis, tumor reduction ratio during external beam radiotherapy (EBRT) with central-shielding method, and cumulative minimal dose to 90% of the high-risk clinical target volume (CTV_HR_ D_90_) on combined EBRT and image-guided adaptive brachytherapy (IGABT) for cervical cancer.

**Methods:**

Consecutive patients who underwent definitive radiotherapy or concurrent chemoradiotherapy for cervical cancer at Gunma University Hospital between January 2010 and December 2019 were retrospectively reviewed. Tumor volume at diagnosis and reduction ratio were calculated using magnetic resonance imaging at diagnosis and before the first IGABT session. The cumulative dose of EBRT and IGABT was calculated as an equivalent dose in 2 Gy fractions (EQD2). Optimal cutoff values were determined according to a receiver operating characteristic curve. Treatment outcomes were evaluated using the Kaplan–Meier method and compared using the log-rank test and Cox proportional hazards regression.

**Results:**

A total of 254 patients were included in the analysis. The median follow-up for all patients was 57 (2–134) months. The 5-year overall survival (OS) was 81.9%, progression-free survival (PFS) was 71.3%, and local control (LC) was 94.5%. The patients were divided into four groups according to tumor volume at diagnosis and reduction ratio. The group with tumor volume at diagnosis ≥ 34.1 cm^3^ and reduction ratio < 68.8% showed significantly worse OS, PFS, and LC than the other three groups (All *p* < 0.05). In this group, the patients with a cumulative CTV_HR_ D_90_ < 69.6 Gy_EQD2_ showed significantly worse PFS and LC (*p* = 0.042 and *p* = 0.027, respectively). In the multivariate analysis of OS, adenocarcinoma/adenosquamous carcinoma, International Federation of Gynecology and Obstetrics 2009 stage III/IV, and a reduction ratio of < 68.8% were independent significant poor prognostic factors (*p* = 0.045, *p* = 0.009 and *p* = 0.001, respectively). In the univariate analysis of LC, a reduction ratio of < 68.8% was the only poor prognostic factor (*p* = 0.041).

**Conclusion:**

The patients with large and poorly responding tumors had significantly worse prognoses in terms of OS, PFS, and LC, suggesting that dose escalation should be considered for such tumors.

## 1 Introduction

The combination of external beam radiotherapy (EBRT) and brachytherapy is the standard treatment of locally advanced cervical cancer (1, 2). Regarding brachytherapy, image-guided adaptive brachytherapy (IGABT) using magnetic resonance imaging (MRI) or computed tomography (CT) has become widespread, resulting in favorable treatment outcomes (3, 4).

Recently, tumor reduction during EBRT has been reported to be a prognostic factor (5–7). Tumor volume at diagnosis and its reduction during EBRT could be crucial factors in appropriate dose delivery in IGABT. Therefore, the prognostic effect should be evaluated in combination of tumor volume at diagnosis, its reduction during EBRT, and cumulative doses of EBRT and IGABT.

In Japan and several other Asian countries, a standard treatment schedule comprises whole-pelvic radiotherapy (WP), followed by central-shielding radiotherapy (CS) and brachytherapy (8, 9). In this treatment schedule, brachytherapy is initiated simultaneously with CS. Therefore, tumor reduction during EBRT is evaluated earlier compared to treatment schedules without CS, which are mainly employed in Europe and the United States. However, the prognostic effect of tumor reduction during EBRT remains unclear.

Thus, the present study aimed to elucidate the prognostic effect of tumor volume at diagnosis, tumor reduction ratio during EBRT, and cumulative minimal dose to 90% of the high-risk clinical target volume (CTV_HR_ D_90_) on combined EBRT with CS method and IGABT for cervical cancer.

## 2 Materials and methods

### 2.1 Patient selection

The clinical records of consecutive patients who underwent definitive radiotherapy or concurrent chemoradiotherapy for cervical cancer at Gunma University Hospital between January 2010 and December 2019 were retrospectively reviewed. The eligibility criteria were: (i) biopsy-proven squamous cell carcinoma, adenocarcinoma (AC), or adenosquamous carcinoma (ASC) of the cervix; (ii) stage IB1–IVA, according to the International Federation of Gynecology and Obstetrics (FIGO) 2009; (iii) patients treated with a combination of EBRT and IGABT; and (iv) tumor volume at diagnosis, reduction ratio, and cumulative CTV_HR_ D_90_ could be evaluated. The study protocol was approved by the Ethics Review Board Committee of Gunma University Hospital (HS2019-226).

### 2.2 External beam radiotherapy

The treatment schedule was in accordance with the Japanese guidelines for the treatment of cervical cancer (10, 11). Three representative treatment schedules are shown in **Supplementary Figure S1**. EBRT was performed using three-dimensional (3D) conformal radiotherapy at a dose of 2 Gy per fraction. WP was delivered using the four-field box technique, including the cervical tumor, uterus, parametrium, upper half of the vagina, and pelvic lymph node regions. The total dose of WP was determined according to FIGO stage and tumor diameter at diagnosis; 20 Gy for FIGO stage I–II tumors ≤ 4 cm, 30 Gy for FIGO stage I–II tumors > 4 cm or FIGO stage III–IV tumors, or up to 40 Gy for bulky extensive tumors. Thereafter, CS was performed with anteroposterior and posteroanterior ports. Using a multi-leaf collimator (MLC), a 3 cm-wide shield was created in the center of the irradiation field with the sacroiliac joint as the upper border. During CS, the dose coverage for the pelvic lymph node regions was evaluated carefully to ensure that the prescribed dose was administered. The CS dose was adjusted to a total of 50 Gy in combination with WP. In patients with lymph node metastases, boost EBRT of 6–10 Gy was administered.

When para-aortic lymph node metastases were present, an extended field was used, and a reduction to 1.8 Gy per fraction was considered. Considering CS inappropriate (due to field size, MLC range of motion, or location of pelvic lymph node metastases), WP of 40 Gy followed by a boost EBRT of 16–18 Gy was allowed.

### 2.3 Image-guided adaptive brachytherapy

Along with CS, CT-based IGABT was performed once a week. The number of sessions was four for tumors treated with 20–30 Gy of WP and three to four for tumors treated with 40 Gy of WP. Additional sessions were considered for AC/ASC or poorly responsive tumors. The tumor response and extent of residual disease were carefully evaluated through gynecologic examination. MRI was performed within 1 week before IGABT as close as possible to the IGABT. For cases treated with 20 Gy of WP, MRI was performed 1–2 days before IGABT to maximize the efficacy of the assessment of tumor responses to WP. The intracavitary/interstitial (IC/IS) technique was recommended for bulky or irregularly shaped tumors (12). CT with the applicator inserted was performed during each IGABT session. The CTV_HR_ was contoured according to the Japanese Radiation Oncology Study Group recommendations (13). Optimization was performed to deliver as much dose as possible to the CTV_HR_ while meeting the following dose constraints for the organs at risk.

The cumulative dose of WP and IGABT was calculated as an equivalent dose in 2 Gy fractions (EQD2) using an α/β of 10 Gy for CTV_HR_ and 3 Gy for organs at risk. The CS dose was excluded from the cumulative dose (14, 15) because the CTV_HR_ D_90_ for the plan comprising intentional exclusion of the CTV_HR_ from the irradiation field does not make sense. The target dose and dose constraints were: cumulative CTV_HR_ D_90_ > 60 Gy_EQD2_; cumulative rectal D_2 cm3_ (minimal dose to the most exposed 2 cm^3^ of the respective organ) < 75 Gy_EQD2_; cumulative bladder D_2 cm3_ < 90 Gy_EQD2_; and cumulative sigmoid D_2 cm3_ < 75 Gy_EQD2_.

### 2.4 Concurrent chemotherapy

Cisplatin-based concurrent chemotherapy was administered to patients with FIGO stage I–II tumors > 4 cm, stage III–IV tumors, or lymph node metastases. Patients aged > 75 years or with severe comorbidities (renal or bone marrow dysfunction) were excluded from receiving concurrent chemotherapy. The eligible patients received up to five courses of weekly cisplatin-based concurrent chemotherapy (40 mg/m^2^).

### 2.5 Follow-up

The patients were followed-up for at least 5 years according to the following schedule, every 1–3 months for the first 2 years of treatment, and then every 3–6 months for the next 3 years. Tumor status and late toxicity were assessed based on patient history; physical examination; and appropriate laboratory and imaging studies. Late toxicity was evaluated according to the Common Terminology Criteria for Adverse Events, version 4.0.

### 2.6 Tumor volume and reduction ratio

Tumor volume was measured using MRI at diagnosis and before the first IGABT session. Three diameters were measured: craniocaudal diameter (ccd) along the axis of the endometrial cavity on the sagittal images; anteroposterior diameter (apd) orthogonal to the ccd; and lateral diameter (ld) measured on the axial images. Tumor volume and reduction ratio were calculated by the following equations: tumor volume = ccd × apd × ld × π/6 and reduction ratio = (tumor volume at diagnosis − tumor volume before IGABT)/tumor volume at diagnosis.

### 2.7 Statistical analysis

Optimal cutoff values for tumor volume at diagnosis, reduction ratio, and cumulative CTV_HR_ D_90_ were determined according to a receiver operating characteristic (ROC) curve using the Youden index, maximizing the sum of sensitivity and specificity for local recurrence. Treatment outcomes were evaluated using the Kaplan–Meier method and compared using the log-rank test and Cox proportional hazards regression. Correlations between continuous variables were evaluated using Spearman’s rank correlation coefficients. Group differences in continuous variables were evaluated using the Kruskal–Wallis test and Dunn’s tests. A *p* value < 0.05 was considered statistically significant. GraphPad Prism version 9 (GraphPad Software, CA, USA) and SPSS Statistics ver. 25 (IBM, NY, USA) were used for analyses.

## 3 Results

### 3.1 Patient characteristics

Between January 2010 and December 2019, 279 consecutive patients underwent definitive radiotherapy or concurrent chemoradiotherapy at Gunma University Hospital. Of these, seven, four, two, and 12 patients: with different histology, with distant metastases, treated with IGABT alone, and without tumor volume or dose information were excluded, respectively. Thus, 254 patients were included in the analysis.

The patient and treatment characteristics are summarized in **Tables 1**, **2**. Pelvic and para-aortic lymph node metastases were observed in 116 and 30 patients, respectively. The total dose of WP was 19.8–20 Gy in 37 patients (14.6%), 30–30.6 Gy in 175 (68.9%), and 38–40 Gy in 42 (16.5%). CS was performed in 248 (97.6%) patients. IGABT was performed three times in seven patients (2.8%), four times in 228 (89.8%), and five times in 19 (7.5%). The patients who underwent IGABT with an IC/IS, at least once, were included in the IC/IS group.

**Table 1:** Patient characteristics.

Characteristics	Values	
Age (years)	60	(27–92)
Histology		
Sq	223	(87.8%)
AC/ASC	31	(12.2%)
FIGO stage		
IB1	24	(9.4%)
IB2	18	(7.1%)
IIA1	9	(3.5%)
IIA2	8	(3.1%)
IIB	94	(37.0%)
IIIA	5	(2.0%)
IIIB	76	(29.9%)
IVA	20	(7.9%)
Nodal status		
Negative	136	(53.5%)
Positive	118	(46.5%)
Tumor volume at diagnosis (cm^3^)	41.6	(0.5–499.5)
Tumor volume before IGABT (cm^3^)	12.9	(0–274.2)
Reduction ratio (%)	65.4	(−120.5–100)

Values are presented as median (range) or number (%).

Sq, squamous cell carcinoma; AC, adenocarcinoma; ASC, adenosquamous carcinoma; FIGO, International Federation of Gynecology and Obstetrics 2009; IGABT, image-guided adaptive brachytherapy.

**Table 2:** Treatment characteristics.

Characteristics	Values	
EBRT dose (Gy)		
Whole pelvic	30	(19.8–40)
Central shielding	20	(0–30.6)
Nodal boost	8	(0–18)
Concurrent chemotherapy		
No	91	(35.8%)
Yes	163	(64.2%)
IGABT technique		
Intracavitary	118	(46.5%)
Intracavitary/Interstitial	136	(53.5%)
CTV_HR_ volume at 1st IGABT (cm^3^)	34.4	(7.7–333.3)
Cumulative CTV_HR_ D_90_ (Gy_EQD2_)	68.9	(47.1–92.2)
Overall treatment time (days)	46.5	(39–77)

Values are presented as median (range) or number (%).

EBRT, external-beam radiotherapy; IGABT, image-guided adaptive brachytherapy; CTV_HR_, high-risk clinical target volume; D_90_, minimal dose to 90% of the target volume; EQD2, equivalent dose in 2 Gy fractions.

### 3.2 Treatment outcomes

The median follow-up in all patients was 57 (2–134) months. The 5-year overall survival (OS) was 81.9%, progression-free survival (PFS) was 71.3%, and local control (LC) was 94.5%. Thirteen (5.1%) patients developed local recurrence, 11 (4.3%) developed pelvic lymph node recurrence, 57 (22.4%) developed distant metastases, and 45 (17.7%) died from any cause. Five patients with local recurrence underwent reirradiation with IC/IS IGABT. The patients with oligometastatic disease were treated with definitive EBRT.

In terms of late toxicity, five (2.0%) patients developed grade 3–4 rectal or sigmoid colon toxicity, and two (0.8%) developed grade 3 bladder toxicity, one of which was the same patient. These included two cases of rectal bleeding, two of rectal or sigmoid colon stenosis or obstruction, one of rectovaginal fistula, one of bladder bleeding, and one of vesicovaginal fistula. The patient who developed a rectovaginal fistula was treated with carboplatin, paclitaxel, and bevacizumab for distant metastases, whereas the patient who developed a vesicovaginal fistula had bladder invasion at diagnosis. No other grade 3 or higher late toxicity was observed.

### 3.3 Tumor volume at diagnosis, reduction ratio, and cumulative CTV_HR_ D_90_

**Figure 1:**
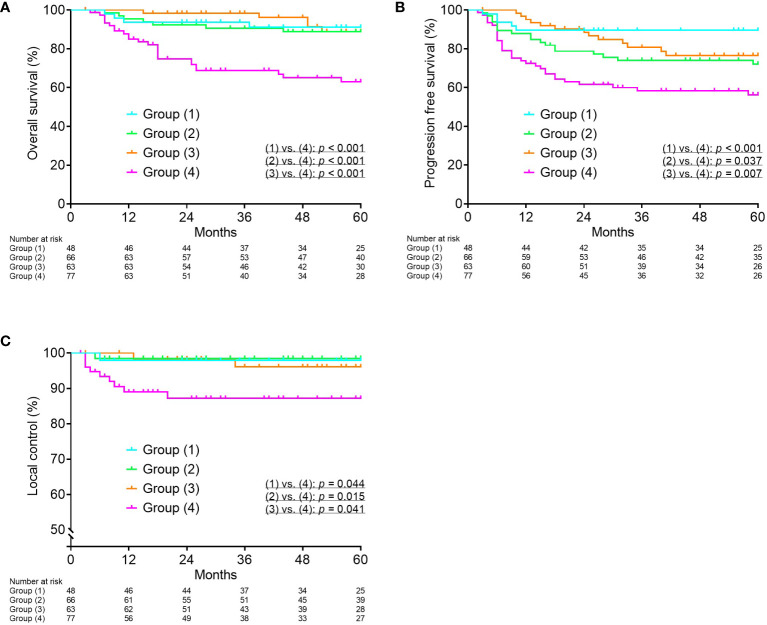
Kaplan–Meier curves for overall survival **(A)**, progression-free survival **(B)**, and local control **(C)** according to tumor volume at diagnosis and reduction ratio. Group (1), tumor volume at diagnosis < 34.1 cm^3^ and reduction ratio ≥ 68.8%; Group (2), tumor volume at diagnosis ≥ 34.1 cm^3^ and reduction ratio ≥ 68.8%; Group (3), tumor volume at diagnosis < 34.1 cm^3^ and reduction ratio < 68.8%; Group (4), tumor volume at diagnosis ≥ 34.1 cm^3^ and reduction ratio < 68.8%.

Based on the ROC curves, the optimal cutoff values for tumor volume at diagnosis, reduction ratio, and cumulative CTV_HR_ D_90_ were determined to be 34.1 cm^3^, 68.8%, and 69.6 Gy_EQD2_, respectively. OS and PFS were significantly worse in the group with tumor volume at diagnosis ≥ 34.1 cm^3^ (*p* = 0.003 and *p* = 0.001, respectively). OS, PFS, and LC were significantly worse in the group with a reduction ratio < 68.8% (*p* = 0.001, *p* = 0.012, and *p* = 0.024, respectively). The patients were divided into four groups according to tumor volume at diagnosis and reduction ratio: group 1, tumor volume at diagnosis < 34.1 cm^3^ and reduction ratio ≥ 68.8%; group 2, tumor volume at diagnosis ≥ 34.1 cm^3^ and reduction ratio ≥ 68.8%; group 3, tumor volume at diagnosis < 34.1 cm^3^ and reduction ratio < 68.8%; and group 4, tumor volume at diagnosis ≥ 34.1 cm^3^ and reduction ratio < 68.8%. Group 4 showed significantly worse OS, PFS, and LC compared to the other three groups (**Figure 1**).

**Figure 2:**
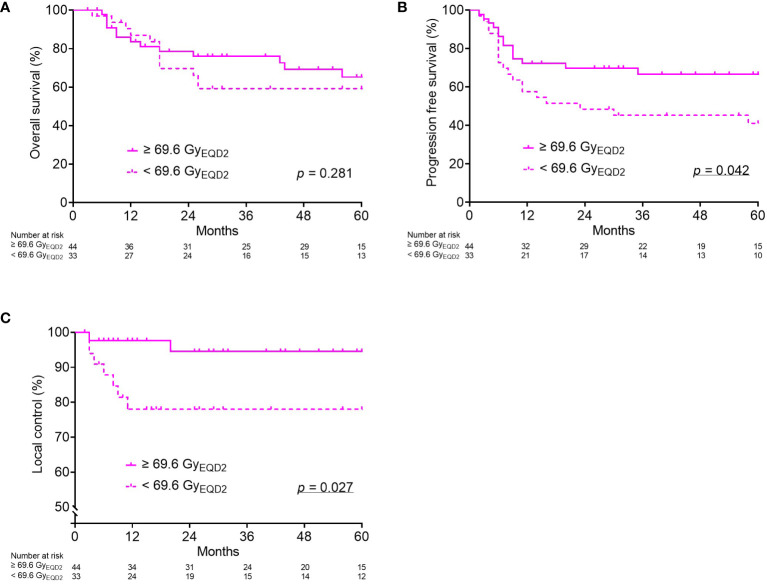
Kaplan–Meier curves for overall survival **(A)**, progression-free survival **(B)**, and local control **(C)** according to cumulative CTV_HR_ D_90_ (Gy_EQD2_) only for Group (4): tumor volume at diagnosis ≥ 34.1 cm^3^ and reduction ratio < 68.8%. CTV_HR_, high-risk clinical target volume; D_90_, minimal dose to 90% of the target volume; EQD2, equivalent dose in 2 Gy fractions.

Thereafter, the dose-response relationship for OS, PFS, and LC was analyzed in each group. The PFS and LC in group 4 were significantly worse in the patients with a cumulative CTV_HR_ D_90_ < 69.6 Gy_EQD2_ (**Figure 2**). In the remaining three groups, there was no significant correlation between the cumulative CTV_HR_ D_90_ and prognosis.

**Figure 3:**
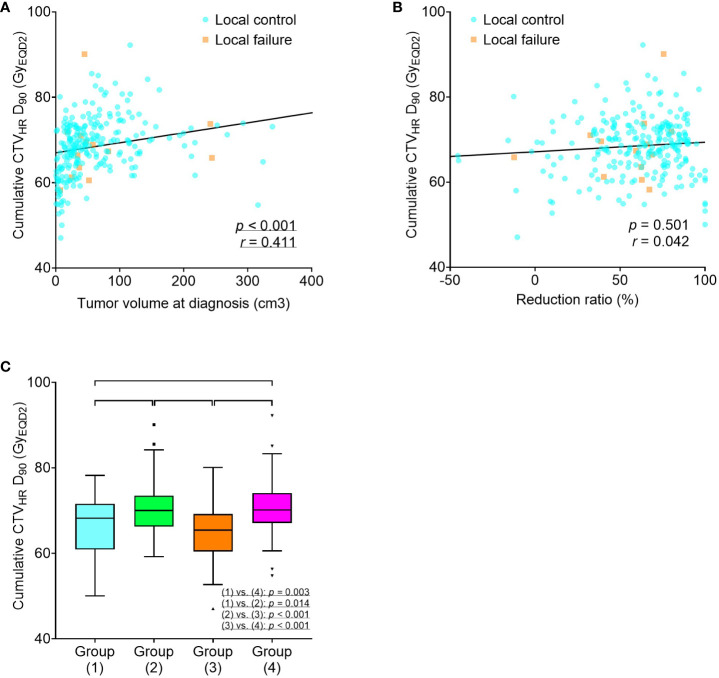
Scatter plots for cumulative CTV_HR_ D_90_ (Gy_EQD2_), tumor volume at diagnosis **(A)**, and reduction ratio **(B)**. Box-and-whisker plots for cumulative CTV_HR_ D_90_ (Gy_EQD2_) according to tumor volume at diagnosis and reduction ratio **(C)**. Group (1), tumor volume at diagnosis < 34.1 cm^3^ and reduction ratio ≥ 68.8%; Group (2), tumor volume at diagnosis ≥ 34.1 cm^3^ and reduction ratio ≥ 68.8%; Group (3), tumor volume at diagnosis < 34.1 cm^3^ and reduction ratio < 68.8%; Group (4), tumor volume at diagnosis ≥ 34.1 cm^3^ and reduction ratio < 68.8%. CTV_HR_, high-risk clinical target volume; D_90_, minimal dose to 90% of the target volume; EQD2, equivalent dose in 2 Gy fractions.

In addition, the relationships between tumor volume at diagnosis, reduction ratio, and cumulative CTV_HR_ D_90_ were analyzed. A positive correlation was found between tumor volume at diagnosis and cumulative CTV_HR_ D_90_, whereas no significant correlation was found between the reduction ratio and cumulative CTV_HR_ D_90_. In the four groups, according to tumor volume at diagnosis and reduction ratio, significant differences were observed in the cumulative CTV_HR_ D_90_, which was higher in the two groups with larger tumor volumes (**Figure 3**).

### 3.4 Univariate and multivariate analyses

**Figure 4:**
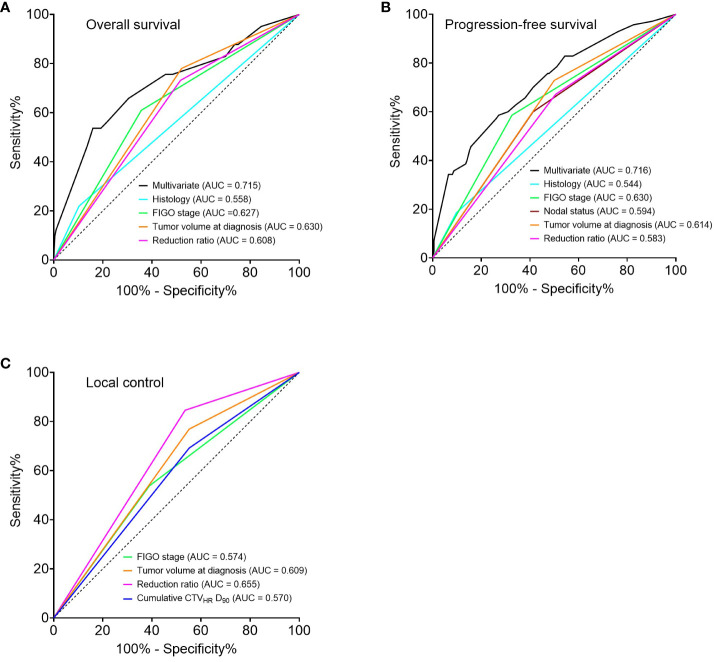
Receiver operator characteristic curves for 5-year prediction of overall survival **(A)**, progression-free survival **(B)**, and local control **(C)** based on univariate and multivariate models. Multivariate analysis was not performed for local control because of the small number of events. FIGO, International Federation of Gynecology and Obstetrics 2009; CTV_HR_, high-risk clinical target volume; D_90_, minimal dose to 90% of the target volume.

Regarding the univariate analysis of OS, AC/ASC histology, FIGO stage III/IV, tumor volume at diagnosis ≥ 34.1 cm^3^, and reduction ratio < 68.8 Gy_EQD2_ were significant poor prognostic factors. Fitting these factors into multivariate analysis, AC/ASC histology, FIGO stage III/IV, and a reduction ratio of < 68.8% were independent significant poor prognostic factors (**Table 3**). The same was true for the PFS (*p* = 0.030, *p* = 0.006 and *p* = 0.008, respectively) (**Supplementary Table S1**). In terms of LC, a reduction ratio of < 68.8% was the only poor prognostic factor using the univariate analysis (*p* = 0.041), and multivariate analysis was not performed because of the small number of events (**Supplementary Table S2**). Additionally, ROC analyses of the univariable and multivariable models were performed. The areas under the curve (AUC) for the multivariate models of OS and PFS were higher than those for the univariate models of OS and PFS. The AUC for the univariate models of the reduction ratio for OS, PFS, and LC were comparable to those for the other univariate models (**Figure 4**).

**Table 3:** Cox regression analysis of overall survival and prognostic factors.

Variables	Univariate HR (95% CI)	*p*-value	Multivariate HR (95% CI)	*p*-value
Age (< 60 years vs. ≥ 60 years)	1.350 (0.746–2.440)	0.321		
Histology (Sq vs. AC/ASC)	2.082 (1.001–4.329)	0.050	2.146 (1.018–4.521)	0.045
FIGO stage (I/II vs. III/IV)	2.702 (1.474–4.954)	0.001	2.414 (1.247–4.675)	0.009
Nodal status (negative vs. positive)	1.764 (0.970–3.207)	0.063		
Tumor volume at diagnosis (< 34.1 cm^3^ vs. ≥ 34.1 cm^3^)	2.654 (1.344–5.238)	0.005	1.962 (0.944–4.080)	0.071
Reduction ratio (≥ 68.8% vs. < 68.8%)	2.908 (1.472–5.746)	0.002	3.103 (1.565–6.152)	0.001
Cumulative CTV_HR_ D_90_ (≥ 69.6 Gy_EQD2_ vs. < 69.6 Gy_EQD2_)	0.823 (0.458–1.480)	0.516		
Concurrent chemotherapy (yes vs. no)	0.965 (0.518–1.796)	0.910		
Overall treatment time (< 56 days vs. ≥ 56 days)	1.607 (0.575–4.490)	0.365		

*p*-values <0.05 are highlighted in underline. HR, hazard ratio; CI, confidence interval; Sq, squamous cell carcinoma; AC, adenocarcinoma; ASC, adenosquamous carcinoma; FIGO, International Federation of Gynecology and Obstetrics 2009; CTV_HR_, high-risk clinical target volume; D_90_, minimal dose to 90% of the target volume; EQD2, equivalent dose in 2 Gy fractions.

## 4 Discussion

The present study showed that tumor volume at diagnosis and the tumor reduction ratio during EBRT were significant prognostic factors for radiotherapy with CS for cervical cancer. In particular, the combination of a large tumor volume at diagnosis and a low tumor reduction ratio during EBRT identified a notably poor prognosis group, and a significant dose-response relationship was observed between cumulative CTV_HR_ D_90_ and PFS or LC in this group, suggesting that dose escalation may be necessary.

Several studies have shown that tumor reduction determined on MRI during EBRT was a significant prognostic factor (6, 7, 16, 17). Most of these studies used treatment without CS and reported a cutoff value of 80–90% tumor volume reduction during EBRT of 45–50 Gy. On the other hand, although reports on treatment with CS are limited, Murakami et al. classified tumors with reduction ratio in diameter ≥ 29% during WP of 20–50 Gy as a low-risk group (18, 19). Okonogi et al. reported significantly better OS, LC, and PFS in patients with AC/ASC in a group with a reduction ratio in diameter > 26.3% during a WP of 20–50 Gy (15). In the present study, a 68.8% reduction in tumor volume was used as the cutoff value during WP of 20–40 Gy, and the reduction ratio was significantly correlated with OS, PFS, and LC. The lower values compared to treatment without CS could reflect the earlier timing of the imaging evaluation and lower dose to tumor. Thus, the tumor reduction ratio during EBRT may be a beneficial prognostic factor, with or without the use of CS.

Few studies have demonstrated the benefits of dose escalation for large volumes at diagnosis and poorly responding tumors. In the present study, a higher cumulative CTV_HR_ D_90_ was associated with significantly better PFS and LC only for this group. Therefore, dose escalation in this group may have contributed to improved outcomes. Due to the low incidence of late toxicities ≥ G3 in the present study, there may be a room for dose escalation by continuation of WP irradiation without CS or additional IGABT sessions. However, there was no correlation between the cumulative CTV_HR_ D_90_ and treatment outcomes in the remaining three groups, all of which achieved local control rates of 95% or higher. The possibility of a cure at low doses has been demonstrated in the low-risk group with small tumor size (19). Therefore, we do not suggest that these three groups could benefit from dose escalation.

There was no significant correlation between the cumulative CTV_HR_ D_90_ and OS, even in group 4. One possible reason for this could be that distant metastasis was the most frequent recurrence pattern and the absolute number of local recurrences was small. Another possible reason might be that salvage treatment was actively administered for local recurrence in the present study. Therefore, further development of systemic therapy in combination with chemoradiotherapy is required to improve OS. The Outback trial (20) assessed the efficacy of adjuvant chemotherapy after definitive chemoradiotherapy, without finding any statistically significant difference. The CALLA trial (21) assessed the efficacy of immune checkpoint inhibitors as concomitant and consolidation therapies with definitive chemoradiotherapy but did not result in a statistically significant improvement. In future studies, a newly developed protocol should be established for the patient group with poor prognosis identified in the present study.

In the present study, tumor volume at diagnosis was positively correlated with the cumulative CTV_HR_ D_90_. This correlation may be mainly due to the de-escalation of the EBRT dose using CS earlier in early disease. The higher rate of IGABT using the IC/IS technique may also have contributed to the higher cumulative CTV_HR_ D_90_ in patients with locally advanced disease. A recent report on IGABT has shown local control of 90% or more, regardless of the FIGO stage (3). The treatment outcomes in the present study were comparable, even in the group with a lower cumulative CTV_HR_ D_90_ compared to those receiving chemoradiotherapy without CS.

The lower cumulative doses to the CTV_HR_ resulted from the use of the CS technique, which is recommended by the treatment guideline in Japan (10, 11). This is supported by evidence from the following prospective studies. Toita et al. reported that the outcomes of treatment with CS and those of treatment without CS were comparable despite the lower dose to point A provided by treatment with CS (22, 23). Murakami et al. reported that the local control of low-risk patients was greater than 90% with CTV_HR_ D_90_ doses of 50–60 Gy_EQD2_ (19). Nakagawa et al. reported that treatment with a median CTV_HR_ D_90_ of 64 Gy_EQD2_ for stage I–II cases with small uterus resulted in no local recurrence (24). The treatment outcomes of the present study cohort were comparable to those of the cohorts of the retroEMBRACE and the EMBRACE I. The 5-year LC and OS were 94.5% and 81.9% in the present study, 89% and 65% in the RetroEMBRACE, and 92% and 74% in the EMBRACE I. Grade 3 or higher late adverse events were 2.4% in the present study, 11% in the RetroEMBRACE, and 14.6% in the EMBRACE I (3, 25). The reasons for the comparable outcomes observed with EBRT and IGABT with CS with a lower CTV_HR_ D_90_ have not been fully explored; therefore, further research is necessary. Nevertheless, the following factors are considered as the possible reasons: (i) the use of CT during delineation of CTV_HR_ may result in lateral diameters greater than those observed with the use of MRI during delineation, thus leading to lower CTV_HR_ D_90_ (26, 27), and (ii) the shorter overall treatment time (approximately 6–7 weeks) may contribute to favorable outcomes (28).

Intensity-modulated radiotherapy (IMRT) and volumetric modulated arc therapy (VMAT) can reduce adverse events during treatment (29, 30). Hence, IMRT and VMAT are recommended as postoperative radiotherapy for cervical cancer (10, 11). However, IMRT and VMAT have not been established as standard practice in definitive radiotherapy for cervical cancer in Asian countries, including Japan, where more than half of the cervical cancer cases have been observed (31). This is partly attributable to the uncertainties regarding organ motion during IMRT and VMAT, as well as limited medical resources (10, 11, 32).

In addition to the reduction ratio, AC/ASC histology and FIGO stage III/IV were poor prognostic factors using the multivariate analysis of overall survival. AC/ASC histology has been shown to have a poor prognosis, even in the era of 3D IGABT (15, 33). In the present study, AC/ASC was not a significant poor prognostic factor in LC but was significant in OS and PFS, possibly due to metastatic susceptibility. Although the FIGO stage partially overlaps with tumor volume, it includes tumor localization and infiltration, which may be advantageous for predicting OS. However, there was no significant correlation between LC and the FIGO stage or tumor volume at diagnosis.

There are several limitations to the present study: first, it was a single-center, retrospective study with a heterogeneous background, including FIGO stage and the presence of concurrent chemotherapy. Second, the measurement of tumor volume on MRI could be subjective and may vary, particularly before IGABT. Third, the treatment schedules varied according to FIGO stage and tumor diameter; the WP dose may have affected the reduction ratio, and CT-based IGABT may have underestimated the actual dose delivered to the tumor. Fourth, the absolute number of local recurrences was small and may have lacked statistical power.

## 5 Conclusion

The present study elucidated the prognostic effect of tumor volume at diagnosis, tumor reduction ratio during EBRT, and cumulative CTV_HR_ D_90_ in the treatment with EBRT with CS and CT-based IGABT for cervical cancer. The treatment outcomes were comparable to those reported in recent studies. The patients with large and poorly responding tumors had significantly worse prognoses in terms of OS, PFS, and LC, suggesting that dose escalation should be considered for such tumors.

## Data availability statement

The original contributions presented in the study are included in the article/**Supplementary Material**. Further inquiries can be directed to the corresponding authors.

## Ethics statement

The studies involving humans were approved by the Ethics Review Board Committee of Gunma University Hospital with the approval number HS2019-226. The studies were conducted in accordance with the local legislation and institutional requirements. Written informed consent for participation was not required from the participants or the participants’ legal guardians/next of kin in accordance with the national legislation and institutional requirements.

## Author contributions

TOht: Writing – original draft, Data curation, Formal analysis, Investigation, Visualization. KA: Conceptualization, Methodology, Project administration, Resources, Writing – review & editing. TOi: Conceptualization, Methodology, Project administration, Writing – review & editing. SN: Resources, Writing – review & editing. TK: Resources, Writing – review & editing. KM: Resources, Writing – review & editing. TOhn: Conceptualization, Methodology, Project administration, Supervision, Writing – review & editing.
