# Supplementary figures and images for: The prognostic effect of tumor volume, reduction ratio, and cumulative doses on external beam radiotherapy with central-shielding method and image-guided adaptive brachytherapy for cervical cancer
Source: Front Oncol. 2024 May 7;14:1366777. doi: 10.3389/fonc.2024.1366777 (PMC11106361; doi:10.3389/fonc.2024.1366777)

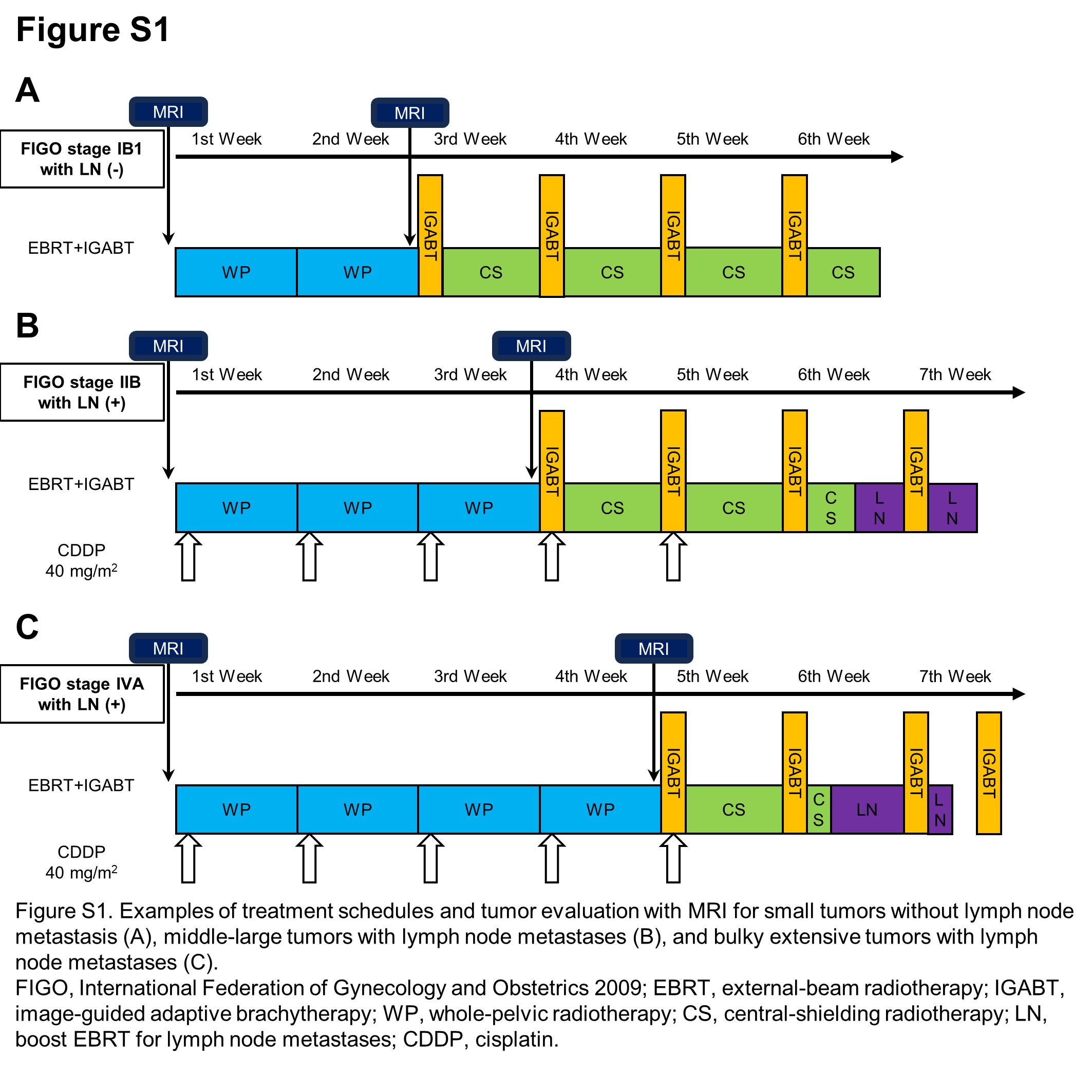

Supplement: Supplementary file 1 [file Image_1.jpeg]
