# Supplementary material for: The prognostic effect of tumor volume, reduction ratio, and cumulative doses on external beam radiotherapy with central-shielding method and image-guided adaptive brachytherapy for cervical cancer
Source: Front Oncol. 2024 May 7;14:1366777. doi: 10.3389/fonc.2024.1366777 (PMC11106361; doi:10.3389/fonc.2024.1366777)
Supplement: Supplementary file 2 [file Table_1.docx]

## Table S1. Cox regression analysis of progression free survival and prognostic factors

| Variables | | Univariate HR (95% CI) | *p*-value | Multivariate HR (95% CI) | *p*-value |
| --- | --- | --- | --- | --- | --- |
| Age (< 60 vs. ≥ 60) | | 1.123 (0.704–1.794) | 0.626 |  |  |
| Histology (Sq vs. AC/ASC) |  | 1.831 (1.002–3.345) | 0.049 | 1.988 (1.068–3.703) | 0.030 |
| FIGO stage (I/II vs. III/IV) | | 2.557 (1.592–4.108) | < 0.001 | 2.140 (1.244–3.679) | 0.006 |
| Nodal status (Negative vs. Positive) | | 1.924 (1.195–3.098) | 0.007 | 1.472 (0.876–2.474) | 0.144 |
| Tumor volume at diagnosis (< 34.1 cm^3^ vs. ≥ 34.1 cm^3^) | | 2.289 (1.365–3.841) | 0.002 | 1.497 (0.838–2.675) | 0.173 |
| Reduction ratio (≥ 68.8% vs. < 68.8%) | | 1.867 (1.135–3.070) | 0.014 | 1.983 (1.199–3.281) | 0.008 |
| Cumulative CTV_HR_ D_90_ (≥ 69.6 Gy_EQD2_ vs. < 69.6 Gy_EQD2_) | | 0.909 (0.569–1.450) | 0.688 |  |  |
| Concurrent chemotherapy (Yes vs. No) | | 0.954 (0.586–1.552) | 0.848 |  |  |
| Overall treatment time (< 56 days vs. ≥ 56 days) | | 1.298 (0.645–2.611) | 0.465 |  |  |

HR, hazard ratio; CI, confidence interval; Sq, squamous cell carcinoma; AC, adenocarcinoma; ASC, adenosquamous carcinoma; FIGO, International Federation of Gynecology and Obstetrics 2009; CTV_HR_, high-risk clinical target volume; D_90_, minimal dose to 90% of the target volume; EQD2, equivalent dose in 2 Gy fractions.

## Table S2. Cox regression analysis of local control and prognostic factors

| Variables | Univariate HR (95% CI) | *p*-value |
| --- | --- | --- |
| Histology (Sq vs. Ad/AdSq) | 0.632 (0.082–4.866) | 0.660 |
| FIGO stage (I/II vs. III/IV) | 1.910 (0.641–5.689) | 0.245 |
| Tumor volume at diagnosis (< 34.1 cm^3^ vs. ≥ 34.1 cm^3^) | 2.813 (0.774–10.228) | 0.116 |
| Reduction ratio (≥ 68.8% vs. < 68.8%) | 4.806 (1.065–21.696) | 0.041 |
| Cumulative CTV_HR_ D_90_ (≥ 69.6 Gy_EQD2_ vs. < 69.6 Gy_EQD2_) | 1.727 (0.532–5.608) | 0.364 |
| Concurrent chemotherapy (Yes vs. No) | 1.105 (0.361–3.378) | 0.861 |
| Overall treatment time (< 56 days vs. ≥ 56 days) | 1.648 (0.365–7.438) | 0.516 |

HR, hazard ratio; CI, confidence interval; Sq, squamous cell carcinoma; AC, adenocarcinoma; ASC, adenosquamous carcinoma; FIGO, International Federation of Gynecology and Obstetrics 2009; CTV_HR_, high-risk clinical target volume; D_90_, minimal dose to 90% of the target volume; EQD2, equivalent dose in 2 Gy fractions.
